# Effects of Resistance Training on Physical Fitness in Healthy Children and Adolescents: An Umbrella Review

**DOI:** 10.1007/s40279-020-01327-3

**Published:** 2020-08-05

**Authors:** Melanie Lesinski, Michael Herz, Alina Schmelcher, Urs Granacher

**Affiliations:** Research Focus Cognition Sciences, Division of Training and Movement Sciences, University of Potsdam, Am Neuen Palais 10, Building 12, 14469 Potsdam, Germany

## Abstract

**Background:**

Over the past decades, an exponential growth has occurred with regards to the number of scientific publications including meta-analyses on youth resistance training (RT). Accordingly, it is timely to summarize findings from meta-analyses in the form of an umbrella review.

**Objectives:**

To systematically review and summarise the findings of published meta-analyses that investigated the effects of RT on physical fitness in children and adolescents.

**Design:**

Systematic umbrella review of meta-analyses.

**Data Sources:**

Meta-analyses were identified using systematic literature searches in the databases PubMed, Web of Science, and Cochrane Library.

**Eligibility Criteria for Selecting Meta-analyses:**

Meta-analyses that examined the effects of RT on physical fitness (e.g., muscle strength, muscle power) in healthy youth (≤ 18 years).

**Results:**

Fourteen meta-analyses were included in this umbrella review. Eleven of these meta-analyses reported between-subject effect sizes which are important to eliminate bias due to growth and maturation. RT produced medium-to-large effects on muscle strength, small-to-large effects on muscle power, small-to-medium effects on linear sprint, a medium effect on agility/change-of-direction speed, small-to-large effects on throwing performance, and a medium effect on sport-specific enhancement. There were few consistent moderating effects of maturation, age, sex, expertise level, or RT type on muscle strength and muscle power across the included meta-analyses. The analysed meta-analyses showed low-to-moderate methodological quality (AMSTAR2) as well as presented evidence of low-to-very low quality (GRADE).

**Conclusion:**

This umbrella review proved the effectiveness of RT in youth on a high evidence level. The magnitude of effects varies according to the respective outcome measure and it appears to follow the principle of training specificity. Larger effect sizes were found for strength-related outcome measures. Future studies should consistently report data on participants’ maturational status. More research is needed with prepubertal children and girls, irrespective of their maturational status.

**Electronic supplementary material:**

The online version of this article (10.1007/s40279-020-01327-3) contains supplementary material, which is available to authorized users.

## Key Points

**Table:** 

Findings from our umbrella review including 14 meta-analyses suggest that RT is an effective means to improve proxies of physical fitness in healthy children and adolescents beyond a level achievable from growth and maturation.
This umbrella review indicates that there are few consistent moderating effects of maturation, age, sex, expertise level, or RT type on muscle strength and muscle power across the included meta-analyses.
This umbrella review identified current gaps in the literature and suggests that future RT research should consistently report data on participants’ maturational status. Pre-pubertal children as well as girls irrespective of their maturational status should be specifically targeted in future research.

## Introduction

Despite previous misconceptions on the effectiveness and safety of youth resistance training (RT), more recent studies show convincing evidence of RT on markers of performance and health in healthy children and adolescents, if appropriately prescribed and supervised [1–3]. In a position statement on youth RT, Lloyd et al. [3] summarised findings from original research, systematic reviews, and meta-analyses and reported that different types of RT (e.g., plyometrics, machine-based RT) have the potential to improve health- (e.g., improved body composition, psychological well-being) and performance-related outcomes (e.g., gains in muscle strength and muscle power). Gathering information from original research in the form of controlled or even randomised-controlled trials is a first step to advance our knowledge in this field of research. Subsequently, findings from original research can be summarised in systematic reviews and statistically aggregated in meta-analyses. However, these publication types (i.e., meta-analyses) are limited in as much as they have a rather narrow focus on one specific outcome measure, a specific population, or a specific RT type. Given these methodological limitations, it is challenging to establish comprehensive recommendations as well as robust pooled results for the overarching topic of youth RT. Further, in-depth literature reviews reveal that there are conflicting results from meta-analyses on youth RT, most likely due to different methodological approaches (e.g., searched databases, search syntax, inclusion criteria, year of literature search) and applied methods (e.g., different statistical methods). For instance, while Lesinski et al. [4] found large effects of RT on muscle power, Collins et al. [5] reported small effects. Furthermore, RT-related effects reported by Lesinski et al. [4] were not moderated by the factor sex. In contrast, Collins et al. [5] observed that the factor sex modulated RT effects on muscle power.

An attempt to overcome the above described methodological limitations of meta-analyses is to perform umbrella reviews [6]. Notably, umbrella reviews are on the highest level of the medicine evidence pyramid [7] and they summarise findings from already published meta-analyses to provide an overview on a given overarching topic. Thus, umbrella reviews help us to understand the current strengths and limitations of the entire body of literature on a specific topic, in this case youth RT.

To the best of our knowledge, there is no published umbrella review available that has examined the effects of RT on measures of physical fitness in healthy children and adolescents.

Therefore, the objectives of this umbrella review were (a) to systematically review the available meta-analytical evidence that has examined the effects of RT on proxies of physical fitness (e.g., muscle strength, muscle power, linear speed) in healthy children and adolescents; (b) to systematically report the effects of potential moderator variables, including maturation, age, sex, expertise level, and RT type (e.g., plyometric training); (c) to address the quality, strengths and limitations of the meta-analytical evidence; and (d) to identify current gaps in the literature and make suggestions for future research.

## Methods

Our umbrella review was conducted in accordance with recommendations for umbrella reviews from Aromataris and colleagues [6] and addressed all items recommended in the PRISMA statement [8].

### Literature Search

We performed a computerized systematic literature search in the databases PubMed, Web of Science, and Cochrane Library. A Boolean search syntax was used (Table 1). The search was limited to full text availability, publication dates from 01/01/1979 to 01/01/2020, ages from birth to 18 years, English and German language, and type of article (i.e., meta-analysis). The reference lists of each included meta-analysis were screened for titles to identify additional meta-analyses to be included in our umbrella review.

**Table 1:** Information on literature search, selection criteria, and considered moderator variables

Literature search	Search syntax	(“strength training“ OR “resistance training“ OR “weight training“ OR “power training“ OR “plyometric training“ OR “complex training“ OR “weight-bearing exercise“) AND (children OR adolescent* OR youth OR puberty OR kid* OR teen* OR girl* OR boy*) AND (“meta-analysis”)
Selection criteria (PICOS)	Population	Healthy young children and adolescents (mean age ≤ 18 years)
	Intervention	RT = a specific method of physical conditioning that involves the progressive use of a wide range of resistive loads, different movement velocities, and a variety of training types (e.g., machine-based RT, free weight RT, elastic bands, plyometrics) [2]
	Comparator	Age-matched control group to avoid bias due to growth and maturation [9], but no alternative RT type as only comparator/control group
	Outcome	At least one measure of muscle strength, muscle power, linear sprint speed, change-of-direction speed/agility, throwing performance, or sport-specific performance
	Study design	Meta-analysis
Potential moderator variables	Chronological age	Children Adolescents
	Maturation status	Prepubertal individuals Mid-/postpubertal individuals according to the maturity offset method (i.e., age at peak-height-velocity) from Mirwald et al. [10] or Tanner stages
	Sex	Boys Girls
	Expertise level	Trained individuals/young athletes Untrained young individuals
	Overall RT types	Traditional RT = conditioning method which involves the use of a wide range of resistive loads and a variety of training types (e.g., machine-based RT, free weights RT) Plyometric training
	Traditional RT types	Machine-based RT Free weights RT

*PICOS* population, intervention, comparator, outcome, study design, *RT* resistance training

### Selection Criteria

Based on a priori defined inclusion/exclusion criteria (PICOS = population, intervention, comparator, outcome, study design; Table 1), two independent reviewers (MH, AS) screened potentially relevant articles by analysing titles, abstracts, and full texts of the respective articles to elucidate their eligibility. When MH and AS did not reach an agreement concerning inclusion of an article, ML adjudicated.

### Data Extraction

The following data were extracted from the included meta-analyses: (1) first author and year of publication; (2) the number and type of primary studies included in the meta-analysis; (3) the study characteristics and the number of included participants; (4) the respective physical fitness outcome; (5) effect sizes and the equations used to compute effect sizes, the respective significance level, *p* values of Chi^2^ tests, the 95% confidence intervals (CI), and *I*^2^ values (i.e., study heterogeneity). Data were extracted and cross-checked for accuracy by ML, MH and AS. If relevant data were not available in the respective papers, we sent email inquiries to the corresponding authors. If the author did not reply or could not provide the missing data, we marked the missing information as “not applicable” (n.a.) or “non-calculable” (n.-c.). Our descriptive analyses focused on different outcome categories (i.e., muscle strength, muscle power, linear sprint speed, agility/change-of-direction speed, throwing performance, and sport-specific performance [e.g., kicking velocity]). Further, we searched the identified meta-analyses for the effects of moderating variables (Table 1).

### Evaluation of the Methodological Quality

Meta-analyses of randomised controlled trials and controlled studies are subject to different sources of bias. Therefore, it is important that readers have the option to distinguish between low and high quality meta-analyses. The methodological quality of the included meta-analyses was independently assessed by three reviewers (ML, MH, and AS) using the validated AMSTAR2 (A Measurement Tool to Assess Systematic Reviews) checklist [11]. This checklist contains 16 items that include for instance the literature search procedure, data extraction, quality assessment, and statistical analyses of the meta-analyses (for more details see [11]). Each item on this checklist was answered with a ‘yes’ (1 point), ‘partial yes’ (0.5 points) or ‘no’ (0 points). Based on the summary point scores (i.e., maximum 16 points), the meta-analyses were categorised as high quality if ≥ 80% of the possible score was achieved, moderate quality if 40–79% of the possible score was reached, or low quality if < 40% of the possible score was achieved [12].

### Quality of Evidence

For the assessment of the quality of evidence, the modified Grading of Recommendations Assessment, Development and Evaluation (GRADE) principles were used [13]. Accordingly, the following GRADE aspects were assessed for each single outcome of the included meta-analyses: (1) study limitations were evaluated through the findings from scales that quantify the risk of bias in the primary studies of the included meta-analyses (e.g., PEDro); (2) inconsistency was assessed through the size of statistical heterogeneity (i.e., *I*^2^-statistics); (3) indirectness was established through the evaluation of differences between study cohorts, intervention types, comparators, and outcome variables of the primary studies and those that were relevant for each included meta-analysis; (4) imprecision was determined using the width of the 95% CI of the pooled effect size of the included meta-analyses; and (5) publication bias was determined by examining the asymmetry of the funnel plots of the included meta-analyses. Each of these five aspects was evaluated for each single outcome as “not reported”, “neutral”, “serious”, or “very serious” [13]. Meta-analyses were downgraded from initially four points by one point for each “not reported” or “serious” and by two points for each “very serious” rating. Then, meta-analyses were rated as “high” (4 points), “moderate” (3 points), “low” (2 points) or “very low” (≤ 1 point) quality of evidence. The GRADE assessment was conducted independently by three authors (ML, MH, and AS), with discussion and agreement regarding any differences.

### Prediction Interval

We calculated the 95% prediction interval (PI) for all included meta-analyses using the number of included studies, the standardised mean difference (SMD), the upper limits of the 95% CI and the tau-squared values (spreadsheet available at: https://www.meta-analysis.com/pages/prediction.php) [14]. The PI represents the range in which the effect size of a future study will most likely fall [14].

### Data Interpretation

The main aims of umbrella reviews is to allow comparison of the magnitude of effects across all included meta-analyses. The use of one effect size measure makes this comparison straightforward. However, it is important to acknowledge that even if most of the included meta-analyses used SMDs as an effect size measure, differences were found in the respective equations that were used to compute SMDs. For instance, some meta-analyses weighted single studies and/or conducted sample size adjustment (e.g., Hedges’ *g*). Therefore, we extracted the equations used to compute effect sizes for each included meta-analysis (Table 2). According to Cohen [15] we classified SMD values < 0.20 as trivial, 0.20 ≤ SMD < 0.50 as small, 0.50 ≤ SMD < 0.80 as medium, and SMD ≥ 0.80 as large effects.

**Table 2:** Included meta-analyses that examined the effects of resistance training on physical fitness in healthy children and adolescents

Study	Population; *N* included participants	N included primary studies; study design; type of RT	Statistical model	Physical fitness outcome	Effect size (95% CI, *p* value); *I*^2^ (Chi^2^ *p* value)	Prediction interval
Meta-analyses reporting between-subject effect sizes						
Behringer et al. [18]	Healthy trained or untrained boys and girls (≤ 18 years) *N* = 1728	*N* = 42 CT and RCT Traditional RT only (no plyometric training)	Between-subject SMD^a,b^ (weighted)	Muscle strength	1.12 (0.9–1.34, *p* < 0.001); 37% (*p* = n.a.) Sub-analyses Maturity (*p* ≤ 0.01) Prepubertal^c^: 0.81 (n.a., *p* = n.a.); n.a. Mid-/postpubertal^d^: 1.91 (n.a., *p* = n.a.); n.a. Sex (*p* ≥ 0.05) Boys: 1.08 (n.a., *p* = n.a.); n.a. Girls: 1.42 (n.a., *p* = n.a.); n.a. Type of RT (*p* ≥ 0.05) Machine-based: 0.93 (n.a., *p* = n.a.); n.a. Free weights: 1.31 (n.a., *p* = n.a.); n.a. Mode of resistance (*p* ≥ 0.05) Isotonic: 1.17 (n.a., *p* = n.a.); n.a. Isokinetic: 1.00 (n.a., *p* = n.a.); n.a. Isometric: 1.03 (n.a., *p* = n.a.); n.a.	1.12 (− 0.44 to 2.68)
Behringer et al. [16]	Healthy trained or untrained boys and girls (≤ 18 years) *N* = 1432	*N* = 34 CT and RCT Any type of RT	Between-subject SMD^a,b^ (weighted)	Jump	0.54 (0.34–0.74, *p* < 0.01); n.a.	n-c
				Linear speed	0.53 (0.23–0.83, *p* < 0.01); n.a.	n-c
				Throw	0.99 (0.19–1.79, *p* < 0.01); n.a.	n-c
				Combined motor performance	0.52 (0.33–0.71, *p* < 0.05); 0% (n.a.) Sub-analyses Expertise (*p* ≥ 0.05) Trained: 0.40 (n.a., *p* = n.a.); n.a. Untrained: 0.64 (n.a., *p* = n.a.); n.a. Type of RT (*p* ≥ 0.05) Plyometric training: 0.51 (n.a., *p* = n.a.); n.a. RT but no plyometric training: 0.54 (n.a., *p* = n.a.); n.a. Mixed (any type of RT): 0.36 (n.a., *p* = n.a.); n.a.	n-c
Collins et al. [5]	Healthy trained or untrained boys and girls (5–18 years) *N* = 943	*N* = 22 CT and RCT Traditional RT only (no plyometric training)	Between-subject SMD^a,e^	Vertical jump	0.41 (0.25–0.56, *p* < 0.001); ≤ 35% (*p* = n.a.)	n-c
				Squat jump	0.73 (0.37–1.09, *p* < 0.001); 59% (*p* = n.a.) Sub-analyses Sex (*p* ≤ 0.01) Boys: 0.84 (0.50–1.18, *p* = n.a.); n.a. Girls: 0.21 (− 0.11 to 0.52, *p* = n.a.); n.a. Expertise (*p* ≤ 0.01) Trained: 0.95 (0.59–1.76, *p* = n.a.); n.a. Untrained: 0.25 (− 0.03 to 0.53, *p* = n.a.); n.a.	n-c
				Standing long jump	0.30 (0.10–0.50, *p* < 0.01); ≤ 35% (*p* = n.a.) Sub-analysis Expertise (*p* ≤ 0.01) Trained: 1.66 (0.75–2.56, *p* = n.a.); n.a. Untrained: 0.23 (0.02–0.43, *p* = n.a.); n.a.	n-c
				Linear speed	0.29 (0.02–0.57, *p* < 0.05); ≤ 35% (*p* = n.a.)	n-c
				Throw	0.41 (0.09–0.72, *p* < 0.05); ≤ 35% (*p* = n.a.)	n-c
Falk et al. [19]	Healthy trained and untrained children (boys: < 13 years, girls: < 12 years) *N* = 635 + n.a.	*N* = 9 CT and RCT Any type of RT	Between-subject SMD^a,b^ (weighted)	Muscle strength	0.57 (0.34–0.80; *p* < 0.001); n.a.	n-c
Harries et al. [24]	Healthy trained boys and girls (13–18 years) *N* = 1070	*N* = 14 CT and RCT Any type of RT	Between-subject mean difference (in cm)^f^ (weighted)	Vertical jump	3.08 cm (1.65–4.51 cm, *p* < 0.001); 68% (*p* < 0.001) Sub-analysis Type of RT (*p* ≥ 0.05) RT, but no plyometric/speed training: 2.09 cm (− 0.01 to 4.20 cm, *p* ≥ 0.05); 38% (*p* > 0.05) Plyometric training: 5.47 cm (1.95–9.00 cm, *p* < 0.01); 48% (*p* > 0.05) RT and plyometric/speed training: 3.03 cm (0.83–5.24 cm, *p* < 0.01); 80% (*p* < 0.001)	3.08 cm (− 2.18 to 8.34 cm)
Lesinski et al. [4]	Healthy trained boys and girls (6–18 years) *N* = 1558	*N* = 43 CT and RCT Any type of RT	Between-subject SMD^a,e^ (weighted)	Muscle strength	1.09 (0.65–1.53, *p* < 0.001); 81% (*p* < 0.001) Sub-analyses Maturity Mid-/postpubertal^g^: 0.61 (0.26–0.96, *p* < 0.001); 18% (*p* > 0.05) Chronological age (*p* ≥ 0.05) Children^h^: 1.35 (0.37–2.33, *p* < 0.01); 68% (*p* < 0.05) Adolescents^i^: 0.91 (0.45–1.37, *p* < 0.001); 78% (*p* < 0.001) Sex Boys: 1.21 (0.64–1.78, *p* < 0.001); 85% (*p* < 0.001) Sport (*p* ≥ 0.05) Team sport: 1.15 (0.64–1.66, *p* < 0.001); 83% (*p* < 0.001) Strength dominated sports: 0.58 (− 0.01 to 1.17, *p* ≥ 0.05); 0% (*p* > 0.05) Type of RT (*p* ≤ 0.001) Machine-based: 0.36 (− 0.44 to 1.16, *p* > 0.05); 62% (*p* > 0.05) Free weights: 2.97 (2.14–3.80, *p* < 0.001); 65% (*p* < 0.05) Machine-based and free weights: 1.16 (0.59–1.73, *p* < 0.001); 40% (*p* > 0.05) Functional training: 0.62 (0.13–1.11, *p* < 0.05); 0% (*p* > 0.05) Plyometric training: 0.39 (0.00–0.77, *p* ≥ 0.05); 0% (*p* > 0.05)	1.09 (− 1.01 to 3.19)
				Vertical jump	0.80 (0.60–1.00, *p* < 0.001); 67% (*p* < 0.001) Sub-analyses Maturity (*p* ≥ 0.05) Prepubertal^j^: 0.91 (0.13–1.69, *p* < 0.05); 80% (p < 0.001) Mid-/postpubertal^g^: 1.15 (0.67–1.64, *p* < 0.001); 82% (*p* < 0.001) Chronological age (*p* ≥ 0.05) Children^h^: 0.78 (0.46–1.10, *p* < 0.001); 63% (*p* < 0.001) Adolescents^i^: 0.85 (0.57–1.13, *p* < 0.001); 70% (*p* < 0.001) Sex (p ≥ 0.05) Boys: 0.85 (0.62–1.07, *p* < 0.001); 69% (*p* < 0.001) Girls: 0.61 (− 0.14 to 1.35, *p* > 0.05); 51% (*p* > 0.05) Sport (*p* ≥ 0.05) Team sport: 0.79 (0.58–1.00, *p* < 0.001); 68% (*p* < 0.001) Strength dominated sports: 1.22 (0.60–1.83, *p* < 0.001); 0% (*p* > 0.05) Type of RT (*p* ≥ 0.05) Machine-based: 1.45 (− 0.33 to 3.22, *p* > 0.05); 90% (*p* < 0.001) Free weights: 0.90 (0.58–1.22, *p* < 0.001); 0% (*p* > 0.05) Machine-based and free weights: 0.77 (0.14–1.41, *p* < 0.05); 45% (*p* > 0.05) Functional training: 0.39 (− 0.09 to 0.88, *p* > 0.05); 26% (*p* > 0.05) Complex training: 1.66 (0.26–3.07, *p* < 0.05); 88% (*p* < 0.001) Plyometric training: 0.81 (0.57–1.06, *p* < 0.001); 60% (*p* < 0.001)	0.80 (− 0.35 to 1.95)
				Linear speed	0.58 (0.41, 0.75, *p* < 0.001); 41% (*p* < 0.01) Sub-analyses Maturity (*p* ≥ 0.05) Prepubertal^j^: 0.65 (0.22–1.08, *p* < 0.01); 42% (*p* > 0.05) Mid-/postpubertal^g^: 0.51 (0.30–0.72, *p* < 0.001); 0% (*p* > 0.05) Chronological age (*p* ≥ 0.05) Children^h^: 0.55 (0.32–0.79, *p* < 0.001); 20% (*p* > 0.05) Adolescents^i^: 0.57 (0.32–0.82, *p* < 0.001); 48% (*p* < 0.05) Sex Boys: 0.63 (0.48–0.78, *p* < 0.001); 16% (*p* > 0.05) Sport Team sport: 0.58 (0.40–0.75, *p* < 0.001); 43% (*p* < 0.01) Type of RT (*p* ≥ 0.05) Free weights: 0.61 (0.30–0.92, *p* < 0.001); 0% (*p* > 0.05) Machine-based and free weights: 0.18 (− 0.34 to 0.69, *p* > 0.05); 0% (*p* > 0.05) Functional training: 0.19 (− 0.51 to 0.89, *p* > 0.05); 64% (*p* > 0.05) Complex training: 1.11 (0.55–1.66, *p* < 0.001); 12% (*p* > 0.05) Plyometric training: 0. (0.41–0.87, *p* < 0.001); 40% (*p* ≥ 0.05)	0.58 (− 0.10 to 1.26)
				Agility/change-of-direction speed	0.68 (0.46–0.90, *p* < 0.001); 50% (*p* < 0.01) Sub-analyses Maturity (*p* ≥ 0.05) Prepubertal^j^: 0.58 (0.13–1.03, *p* < 0.05); 0% (*p* > 0.05) Mid-/postpubertal^g^: 0.57 (0.34–0.80, *p* < 0.001); 0% (*p* > 0.05) Chronological age (*p* ≥ 0.05) Children^h^: 0.52 (0.25–0.78, *p* < 0.001); 17% (*p* > 0.05) Adolescents^i^: 0.71 (0.36–1.06, *p* < 0.001); 62% (*p* < 0.01) Sex Boys: 0.74 (0.52–0.95, *p* < 0.001); 42% (*p* < 0.05) Sport Team sport: 0.68 (0.46–0.90, *p* < 0.001); 50% (*p* < 0.01) Type of RT (*p* ≤ 0.05) Free weights: 1.31 (0.89–1.72, *p* < 0.001); 0% (*p* > 0.05) Functional training: 0.38 (− 0.83 to 1.59, *p* > 0.05); 88% (*p* < 0.001) Complex training: 0.66 (− 0.01 to 1.32, *p* ≥ 0.05); 42% (*p* > 0.05) Plyometric training: 0.62 (0.41–0.83, *p* < 0.001); 19% (*p* > 0.05)	0.68 (− 0.17 to 1.53)
				Sport-specific performance	0.75 (0.48–1.02, *p* < 0.001); 62% (*p* = 0.001) Sub-analyses Maturity (*p* ≥ 0.05) Prepubertal^j^: 0.27 (− 0.17 to 0.72, *p* > 0.05); 0% (*p* > 0.05) Mid-/postpubertal^g^: 0.72 (0.26–1.18, *p* < 0.01.); 64% (*p* < 0.01) Age (*p* ≥ 0.05) Children^h^: 0.5 (0.27–0.72, *p* < 0.001); 0% (*p* > 0.05) Adolescents^i^: 1.03 (0.55–1.51, *p* < 0.001); 73% (*p* < 0.001) Sex (*p* ≤ 0.05) Boys: 0.72 (0.44–1.01, *p* < 0.001); 58% (*p* < 0.001) Girls: 1.81 (0.82–2.80, *p* < 0.001); 33% (*p* > 0.05) Sport (*p* ≥ 0.05) Team sport: 0.80 (0.52–1.09, *p* < 0.001); 61% (*p* < 0.001) Strength dominated sports: 0.34 (− 0.60 to 1.28, *p* > 0.05); 71% (*p* < 0.05) Type of RT (*p* ≤ 0.05) Machine-based: 0.30 (− 0.36 to 0.97, *p* > 0.05); 43% (*p* > 0.05) Functional training: 0.79 (0.15–1.44, *p* < 0.05); 72% (*p* < 0.01) Complex training: 1.85 (1.12–2.58, *p* < 0.001); 0% (*p* > 0.05) Plyometric training: 0.74 (0.39–1.08, *p* < 0.001); 57% (*p* < 0.01)	0.75 (− 0.46 to 1.96)
Moran et al. [26]	Healthy trained boys (10–18 years) *N* = 539	N = 21; CT & RC; Plyometric training only	Between-subject SMD^a,e^ (weighted)	Counter-movement jump	0.73 (0.47–0.99; p < 0.001); 61% (p < 0.001) **Sub-analyses** Age - Children ^k^: 0.91 (0.47–1.36, p > 0.001); 59% (p < 0.01) - Younger adolescents ^l^: 0.47 (0.16–0.77, p < 0.01); 53% (p < 0.05) - Older adolescents ^m^: 1.02 (0.52 –1.53, p < 0.001); 3% (p > 0.05) Sport - Soccer: 0.61 (0.36–0.86, p < 0.001); 45% (p < 0.05) - Other: 1.09 (0.38 –1.80, p < 0.01); 77% (p < 0.01) RT period < 7.5 weeks 0.38 (0.19–0.56, p < 0.001); 0% (p > 0.05) > 7.5 weeks: 1.21 (0.72–1.69, p < 0.001); 71% (p < 0.001) RT sessions < 14.5 sessions: 0.37 (0.19–0.56, p < 0.001); 0% (p > 0.05) > 14.5 sessions: 1.28 (0.78–1.78, p < 0.001); 71% (p > 0.05)	0.73 (− 0.41 to 1.87)
Moran et al. [22]	Healthy trained or untrained girls (8–18 years) *N* = 351	*N* = 11 CT and RCT Any type of RT	Between-subject SMD^a,e^ (weighted)	Muscle strength	0.54 (0.23–0.85, p < 0.001); 42% (p < 0.05) Sub-analyses Age < 15 years: 0.38 (-0.02–0.79, p > 0.05); 41% (p > 0.05) > 15 years: 0.72 (0.23–1.21, p < 0.01); 42% (p > 0.05) Body height < 163 cm: 0.55 (0.08–1.02, p < 0.05); 57% (p < 0.05) > 163 cm: 0.67 (0.20–1.13, p < 0.01); 18% (p > 0.05) Body mass < 56 kg: 0.53 (0–1.06, p ≥ 0.05); 60% (p < 0.05) > 56 kg: 0.67 (0.30–1.03, p < 0.001); 3% (p > 0 05) RT period ≤ 8 weeks: 0.62 (0.17–1.07, p < 0.01); 56% (p < 0.05) > 8 weeks: 0.44 (-0.02–0.90, p > 0.05); 25% (p > 0.05) RT frequency ≤ 2 sessions/week: 0.72 (0.34–1.09, p < 0.001); 36% (p > 0.05) > 2 sessions/week: 0.18 (-0.26–0.61, p > 0.05); 21% (p > 0.05) RT sessions ≤ 16 sessions: 0.75 (0.33–1.17, p < 0.001); 35% (p > 0.05) > 16 sessions: 0.30 (-0.11–0.72, p > 0.05); 33% (p > 0.05) RT duration < 40 min per session: 0.34 (-0.38–1.06, p < 0.05); 53% (p > 0.05) > 40 min per session: 0.63 (0.11–1.16, p < 0.05); 4% (p > 0.05)	0.54 (-0.41-1.49)
Moran et al. [21]	Healthy trained or untrained girls (8-18 years); N = 452	N = 14; CT & RCT; Plyometric training only	Between-subject SMD ^a,e^ (weighted)	Vertical jump	0.57 (0.21–0.93; p < 0.01); 68% (p < 0.001) **Sub-analyses** Age < 15 years: 0.78 (0.25–1.30, p < 0.01); 71% (p < 0.01) > 15 years: 0.31 (-0.18–0.80, p > 0.05); 61% (p < 0.05) Body height < 163 cm: 1.03 (0.38–1.68, p < 0.01); 72% (p < 0.001) ≥ 163 cm: 0.25 (-0.20–0.70, p > 0.05); 63% (p < 0.05) Body mass < 54 kg: 1.14 (0.39–1.89, p < 0.01); 76% (p < 0.001) ≥ 54 kg: 0.26 (-0.15–0.67, p > 0.05); 56% (p < 0 05) RT period ≤ 8 weeks: 0.24 (-0.11–0.59, p > 0.05); 38% (p > 0.05) > 8 weeks: 1.04 (0.35–1.72, p < 0.01); 79% (p < 0.01) RT frequency ≤ 2 sessions/week: 0.37 (0.02–0.71, p < 0.05); 52% (p < 0.01) > 2 sessions/week: 1.22 (0.18–2.25, p < 0.05); 83% (p < 0.01) RT sessions < 16 sessions: 0.37 (-0.44–1.17, p > 0.05); 77% (p > 0.01) = 16 sessions: 0.46 (0.08–0.84, *p* < 0.05); 1% (*p* > 0.05) > 16 sessions: 0.85 (0.18–1.51, *p* < 0.05); 77% (*p* < 0.001) RT duration < 40 min per session: 0.33 (0.03–0.63, *p* < 0.05); 0% (*p* > 0.05) > 40 min per session: 1.16 (0.14–2.17, *p* < 0.05); 76% (*p* < 0.01)	0.57 (-0.81-1.95)
Payne et al. [17]	Healthy trained or untrained boys and girls (11–18 years) *N* = 252	*N* = 28 CT and RCT n.a.	Between-subject SMD^n^ (not weighted)	Muscle strength and endurance	0.75 (n.a., *p* < 0.05); 63% (*p* = n.a.) Sub-analyses Age Children^o^: 0.75 (n.a., *p* < 0.05); 55% (*p* = n.a.) Adolescents^p^: 0.69 (n.a., *p* < 0.05); 79% (*p* = n.a.) Sex Boys: 0.72 (n.a., *p* < 0.05); 57% (*p* = n.a.) Girls: 0.81 (n.a., *p* < 0.05); 69% (*p* = n.a.) Mode of resistance Isokinetic: 0.20 (n.a., *p* < 0.05); 59% (*p* = n.a.) Isometric: 0.71 (n.a., *p* < 0.05); 70% (*p* = n.a.) Isotonic: 0.90 (n.a., *p* < 0.05); 72% (*p* = n.a.) Body segment Arm: 0.74 (n.a., *p* < 0.05); 59% (*p* = n.a.) Back: 0.83 (n.a., *p* < 0.05); 79% (*p* = n.a.) Leg: 0.71 (n.a., *p* < 0.05); 65% (*p* = n.a.)	n-c
Slimani et al. [23]	Healthy trained boys and girls (6–18 years) *N* = 428	*N* = 15 CT and RCT No plyometric or power training	Between-subject SMD^e^ (weighted)	Counter-movement jump	0.65 (0.34–0.96, *p* < 0.001); 53% (*p* = n.a.) Sub-analyses Age (*p* ≥ 0.05) Children^q^: 0.41 (− 0.07 to 0.89, *p* > 0.05); 22% (*p* = n.a.) Adolescents^r^: 0.69 (0.29–1.08, *p* < 0.001); 56% (*p* = n.a.) Sex (*p* ≤ 0.05) Boys: 0.79 (0.43–1.15, *p* < 0.001); 55% (*p* = n.a.) Boys and girls: 0.18 (− 0.24 to 0.60, *p* < 0.05); 0% (*p* = n.a.) Expertise level (*p* ≥ 0.05) Trained: 0.81 (0.38–1.25, *p* < 0.001); 60% (*p* = n.a.) Recreationally trained: 0.36 (0.01–0.72, *p* < 0.05); 0% (*p* = n.a)	n-c
				Squat jump	0.80 (0.23–1.37, *p* < 0.05); 71% (*p* = n.a.) Sub-analyses Age (*p* ≤ 0.05) Children^q^: − 0.54 (− 1.44 to 0.35, *p* > 0.05); 0% (*p* = n.a.) Adolescents^r^: 0.95 (0.40–1.50, *p* < 0.001); 65% (*p* = n.a.) Sex (*p* ≥ 0.05) Boys: 0.89 (0.27–1.51, *p* < 0.01); 72% (*p* = n.a.) Boys and girls: 0.07 (− 0.72 to 0.86, *p* > 0.05); 0% (*p* = n.a.)	n-c
Meta-analyses reporting within-subject effect sizes						
Asadi et al. [27]	Healthy trained or untrained boys (10–18 years) *N* = 669	*N* = 16 CT and RCT Plyometric training only	Within-subject SMD^a,s^	Change-of-direction speed	0.86 (n.a., *p* ≤ 0.05); n.a. Sub-analysis Age Children^k^: 0.68 (n.a., *p* ≤ 0.05); n.a. Younger adolescents^l^: 0.95 (n.a., *p* ≤ 0.05); n.a. Older adolescents^m^: 0.99 (n.a., *p* ≤ 0.05); n.a.	n-c
Behm et al. [20]	Healthy trained or untrained boys and girls (< 18 years) *N* = 1351	*N* = 107 CT and RCT Any type of RT	Within-subject SMD^a,s^ (weighted)	Muscle strength	RT 1.14 (0.89–1.39, *p* < 0.05); 77% (*p* < 0.001) Sub-analyses Expertise (*p* ≥ 0.05) Trained: 1.23 (0.80–1.67, *p* < 0.001); 81% (*p* < 0.001) Untrained: 1.08 (0.78–1.38, *p* < 0.001); 77% (*p* < 0.001) Age (*p* ≥ 0.05) Children: 1.39 (0.89–1.90, *p* < 0.001); 80% (*p* < 0.001) Adolescents: 0.88 (0.61–1.14, *p* < 0.001); 67% (*p* < 0.001)	1.14 (− 0.35 to 2.63)
					Power training 0.16 (− 0.26 to 0.58, *p* > 0.05); 0% (*p* > 0.05)	0.16 (− 2.56 to 2.88)
				Muscle power	RT 0.52 (0.39–0.64, *p* < 0.05); 29% (*p* < 0.05) Sub-analyses Expertise (*p* ≥ 0.05) Trained: 0.48 (0.33–0.63, *p* < 0.001); 13% (*p* > 0.05) Untrained: 0.61 (0.37–0.85, *p* < 0.05); 49% (*p* < 0.001) Age (*p* ≥ 0.05) Children: 0.68 (0.45–0.91, *p* < 0.05); 44% (*p* < 0.05) Adolescents: 0.42 (0.28–0.56, *p* < 0.05); 9% (*p* > 0.05)	0.52 (0.05–0.99)
					Power training 0.69 (0.53–0.84, *p* < 0.001); 51% (*p* < 0.001) Sub-analyses Expertise (*p* ≥ 0.05) Trained: 0.67 (0.52–0.82, *p* < 0.001); 39% (*p* < 0.05) Untrained: 0.80 (0.24–1.35, *p* < 0.01); 80% (*p* < 0.001) Age (*p* ≥ 0.05) Children: 0.74 (0.53–0.94, *p* < 0.001); 62% (*p* < 0.001) Adolescents: 0.57 (0.37–0.77, *p* < 0.001); 14% (*p* > 0.05)	0.69 (− 0.11 to 1.49)
				Linear speed	RT 0.48 (0.25–0.71, *p* < 0.001); 30% (*p* > 0.05) Sub-analyses Expertise (*p* ≥ 0.05) Trained: 0.45 (0.19–0.70, *p* < 0.001); 28% (*p* > 0.05) Untrained: 0.57 (− 0.02 to 1.16, *p* > 0.05); 45% (*p* > 0.05) Age (*p* ≥ 0.05) Children: 0.73 (0.35–1.12, *p* < 0.001); 17% (*p* > 0.05) Adolescents: 0.36 (0.10–0.62, *p* > 0.05); 19% (*p* > 0.05)	0.48 (− 0.15 to 1.11)
					Power training 0.38 (0.23–0.53, *p* < 0.001); 12% (*p* > 0.05) Sub-analyses Expertise (*p* ≥ 0.05) Trained: 0.32 (0.18–0.46, *p* < 0.001); 0% (*p* > 0.05) Untrained: 1.19 (− 0.32 to 2.69, *p* < 0.001); 87% (*p* < 0.001) Age (*p* ≥ 0.05) Children: 0.47 (0.28–0.67, *p* < 0.001); 31% (*p* > 0.05) Adolescents: 0.13 (− 0.17 to 0.44, *p* > 0.05); 0% (*p* > 0.05)	0.38 (0.04–0.72)
Moran et al. [25]	Healthy, trained boys (10–18 years) *N* = 496	*N* = 19 n.a. Any type of RT	Within-subject SMD^a,s^ (weighted)	Muscle strength	0.98 (0.70–1.27, *p* < 0.001); 75% (*p* < 0.001) Sub-analysis Age Children^k^: 0.50 (− 0.06 to 1.07, *p* > 0.05); 54% (*p* > 0.05) Younger adolescents^l^: 1.11 (0.67–1.54, *p* < 0.001); 80% (*p* < 0.001) Older adolescents^m^: 1.01 (0.56 − 1.46, *p* < 0.001); 71% (*p* < 0.001)	0.98 (− 0.51 to 2.47)

*PICOS* population, intervention, comparator, outcome, study design, *RT* resistance training, *CT* controlled trial, *n.a.* not applicable, *n.-c.* not-computable, *p* significance level, *RCT* randomised controlled trial, *RT* resistance training

^a^Standardised mean differences (SMD) were adjusted for the respective sample size using the term 1 − $\frac{3}{4N - 9}$ (Hedges’ *g*)

^b^Between-subject standardised mean differences (SMD) = (mean post value intervention group − mean pre value intervention group) − (mean post value control group − mean pre value control group)/pooled standard deviation)

^c^Tanner stage I

^d^Tanner stage II–V

^e^Between-subject standardised mean differences (SMD) = (mean post value intervention group − mean post value control group)/pooled standard deviation)

^f^Between-subject mean difference (MD) = mean post value intervention group − mean post value control group

^g^Tanner stage III–V

^h^Boys ≤ 13 years; girls ≤ 11 years

^i^Boys 14–18 years; girls 12–18 years

^j^Tanner stage I and II

^k^10–12.99 years

^l^13–15.99 years

^m^16–18 years

^n^Between-subject standardised mean differences (SMD) = (mean post value intervention group − mean post value control group)/post value standard deviation control group)

^o^Boys < 13 years; girls < 11 years

^p^Boys ≥ 16 years; girls ≥ 14 years

^q^Boys 6–13 years; girls 6–11 years

^r^Boys 14–18 years; girls 12–18 years

^s^Within-subject standardised mean differences (SMD = (mean post value intervention group − mean pre value intervention group)/pooled standard deviation)

## Results

### Search Results

**Fig. 1:**
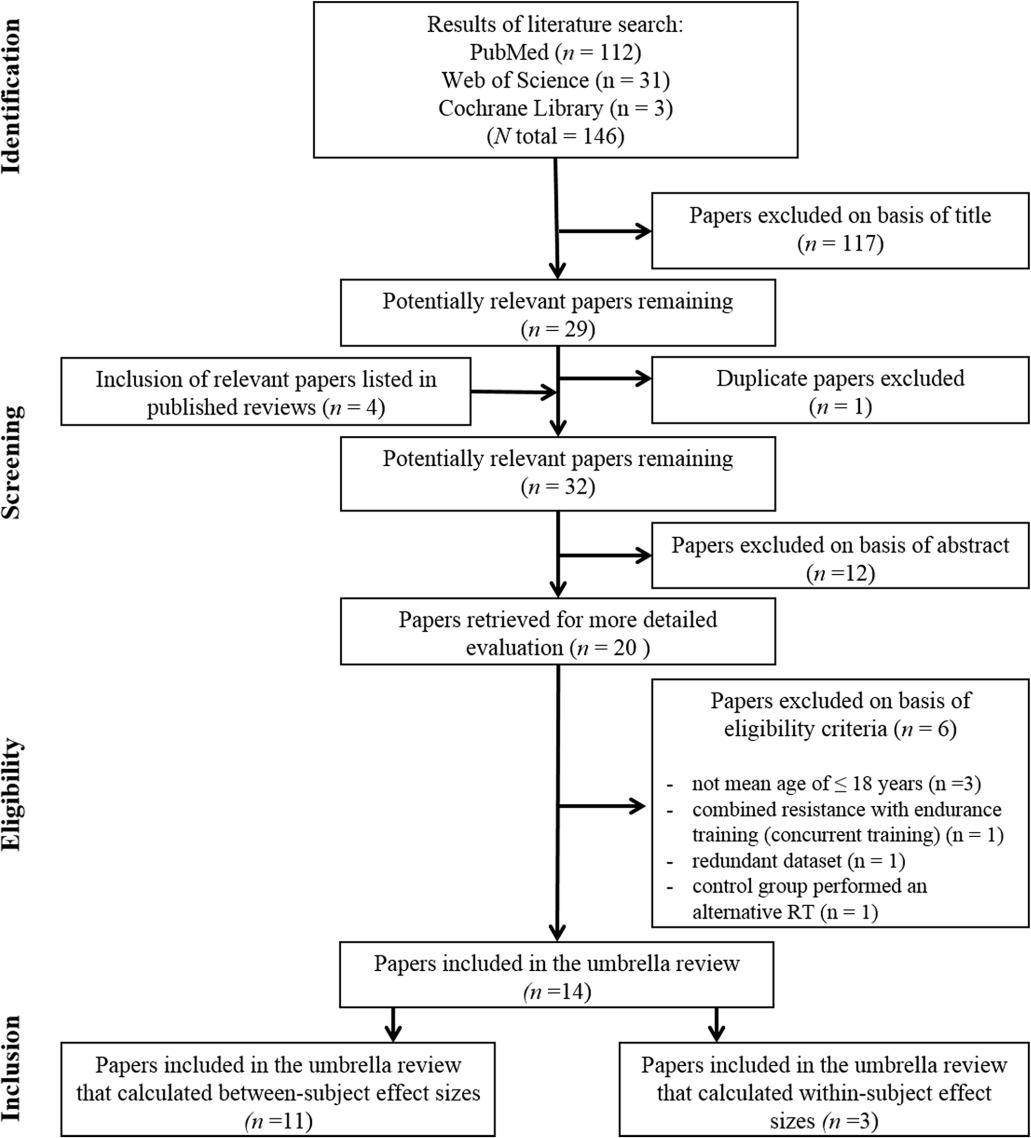
Flow chart illustrating the different phases of the search and study selection

A total of 146 potentially relevant studies were identified in the electronic databases (Fig. 1). Finally, 14 meta-analyses were eligible for inclusion in this umbrella review based on a priori defined selection criteria. We further separated the included meta-analyses into those that reported between-subject effect sizes (i.e., post-test comparison of the intervention versus control group) and those that reported within-subject effect sizes (i.e., pre- versus post-test comparison of the intervention group) (Table 2).

### Characteristics of the Meta-analyses

The 14 included meta-analyses were published between 1996 and 2019. The number of included original studies ranged from nine to 43 with an average of 28 original studies. Sample sizes ranged from 252 to 1728 trained and untrained healthy children and adolescents (average: 847 participants). The chronological age of the included participants ranged from 6 to 18 years. Six meta-analyses investigated the effects of RT in trained and untrained girls and boys,[5, 16–20] two meta-analyses in trained and untrained girls [21, 22], one meta-analysis in trained and untrained boys, three meta-analyses in trained boys and girls [4, 23, 24], and two meta-analyses in trained boys [25, 26]. Regarding the type of RT, eight meta-analyses [4, 16, 17, 19, 20, 22, 24, 25] included any type of RT, three meta-analyses [5, 18, 23] excluded plyometric training, and three meta-analyses [21, 26, 27] specifically focused on plyometric training.

The assessment of the methodological quality (AMSTAR2) of the included meta-analyses was summarised in Electronic Supplementary Material (Table S1). The included papers received scores ranging between 6 and 72% of the maximum score (16 points). Three meta-analyses [4, 5, 16] were of moderate quality and the remaining eleven meta-analyses of low methodological quality. The following criteria were not sufficiently addressed in the analysed meta-analyses: (2) establish methods prior to the conduct of the meta-analyses (written protocol); (3) explain the choice of study design for inclusion; (7) provide a list of excluded studies to justify the exclusion; and (10) report sources of funding for included studies.

The assessment of the quality of evidence (GRADE) of the included meta-analyses was summarised in Electronic Supplementary Material (Table S2). Two of the included meta-analyses [22, 23] presented evidence of low quality and eight meta-analyses [17–19, 21, 24–27] provided evidence of very low quality. The remaining four meta-analyses [4, 5, 16, 20] presented evidence of low to very low quality depending on the outcome measure under consideration.

### Effectiveness of Resistance Training in Healthy Youth

To avoid bias due to growth and maturation-related performance enhancements, we focused only on the included meta-analyses that reported between-subject effect sizes (Table 1).

**Fig. 2:**
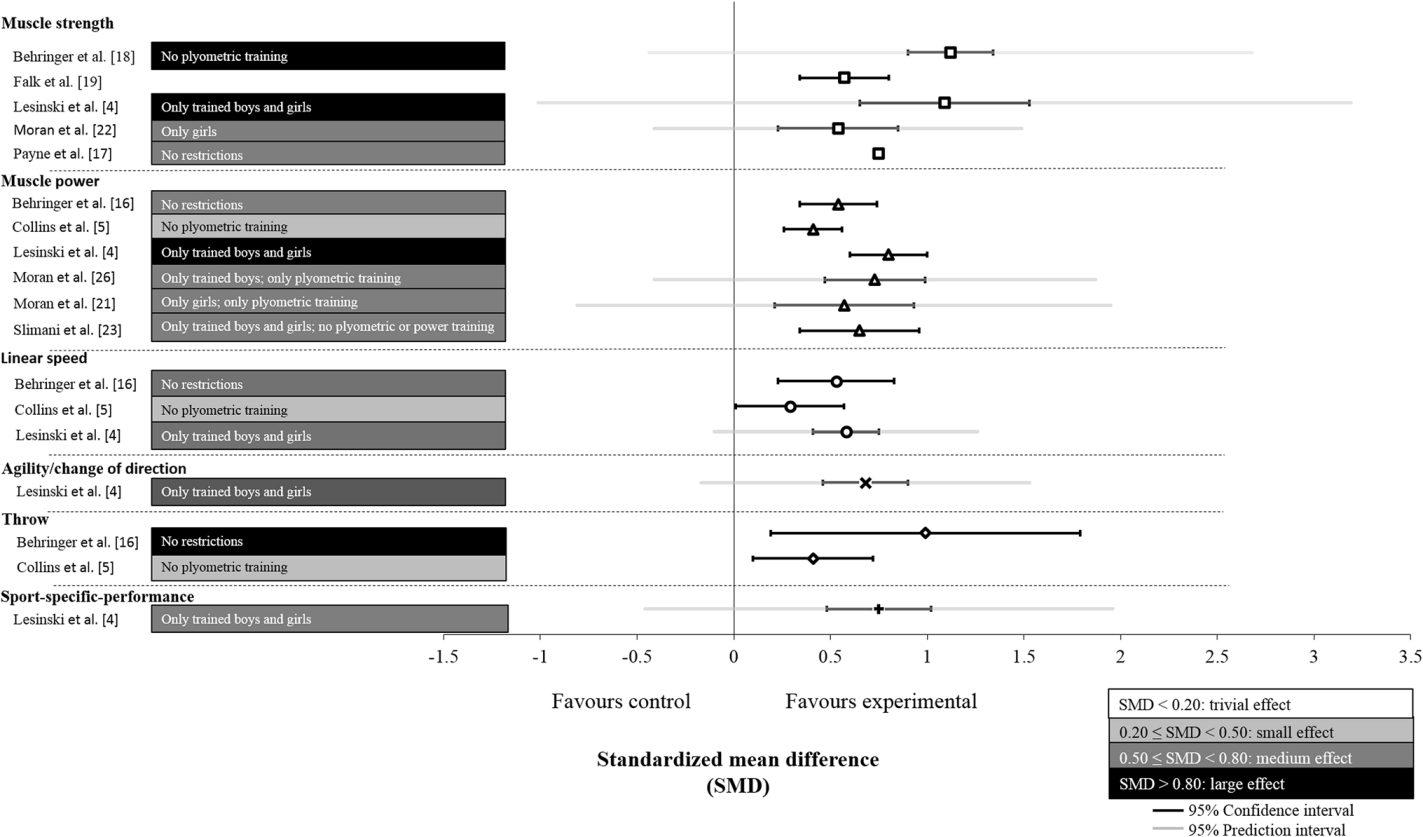
Summary of the effect sizes (between-subject standardised mean difference [SMD]), 95% confidence intervals (black lines), and 95% prediction intervals (grey lines) from the included meta-analyses, indicating the effects of resistance training (RT) versus control group on proxies of physical fitness in healthy children and adolescents. Bars indicate the magnitude of the effects of RT for each meta-analysis including the restriction regarding the included population or type of included RT

The included meta-analyses indicated medium-to-large effects (0.54 ≤ SMD ≤ 1.12) of RT on muscle strength [4, 17, 18, 22], small-to-large effects (0.41 ≤ SMD ≤ 0.80) on muscle power [4, 5, 16, 21, 23, 24, 26], small-to-medium effects (0.30 ≤ SMD ≤ 0.53) on linear speed [4, 5, 16], medium effects (SMD = 0.68) on agility/change-of-direction speed [4], small-to-large effects (0.41 ≤ SMD ≤ 0.99) on throwing performance [5, 16], and medium effects (SMD = 0.75) on sport-specific performance [4] in trained and untrained children and adolescents (Fig. 2).

### Maturation-, Age-, Sex-, Expertise Level- and Type-Specific Effects of Resistance Training on Muscle Strength and Muscle Power

Several of the included meta-analyses performed sub-group analyses of moderator variables which were summarised in Tables 3 and 4.

**Table 3:** Summary of the findings (effect sizes = standardised mean differences) of the sub-group analyses regarding resistance training related effects of the moderator variables age, maturation, sex, and expertise level on measures of muscle strength and muscle power in children and adolescents

Outcomes	Study	Chronological age			Maturational status			Sex			Expertise level		
		Children	Adolescents	*p*	Prepubertal	Mid-/postpubertal	*p*	Boys	Girls	*p*	Trained (athletes)	Untrained	*p*
Muscle strength	Behringer et al. [18]^a^	0.21		> 0.05	0.81 (*n* = n.a./16)	1.91 (*n* = n.a./11)	< 0.05	1.08 (*n* = 29/43)	1.42 (*n* = 8/10)	> 0.05	–		
	Behm et al. [20]^b,c^	1.39 (*n* = 13/14)	0.88 (*n* = 22/27)	> 0.05	–			–			1.23 (*n* = 19/22)	1.08 (*n* = 18/22)	> 0.05
	Falk et al. [19]^a^	0.57 (*n* = 9/n.a.)			–			–			–		
	Lesinski et al. [4]^a^	1.35 (*n* = 3/4)	0.91 (*n* = 13/17)	> 0.05		0.61 (*n* = 6/8)		1.21 (*n* = 12/18)			1.09 (*n* = 16/23)		
	Moran et al. [22]^a^	–			–				0.54 (*n* = 11/16)		–		
	Moran et al. [25]^b^	0.50 (*n* = 3/4)	1.11 (*n* = 11/17) and 1.01* (*n* = 7/11)	> 0.05*	–			0.98 (*n* = 19/32)			0.98 (*n* = 19/32)		
	Payne et al. [17]^a^	0.75 (*n* = n.a.)	0.69 (*n* = n.a.)	n.a.	–			0.72 (*n* = n.a.)	0.81 (*n* = n.a.)	n.a.	–		
Muscle power	Behm et al. [20]^b,c^	0.68 (*n* = 16/17)	0.42 (*n* = 23/30)	> 0.05*	–			–			0.48 (*n* = 23/30)	0.61 (*n* = 14/16)	> 0.05
	Behm et al. [20]^b,d^	0.74 (*n* = 25/34)	0.57 (*n* = 16/20)	> 0.05*	–			–			0.67 (*n* = 33/46)	0.80 (*n* = 6/8)	> 0.05
	Collins et al. [5]^a^ vertical jump	n.a.	n.a.	> 0.05	n.a.	n.a.	> 0.05	–			–		
	Collins et al. [5]^a^ SJ	n.a.	n.a.	> 0.05	n.a.	n.a.	> 0.05	0.84 (*n* = 9/14)	0.21 (*n* = 1/1)	< 0.01	0.95 (*n* = 7/n.a.)	0.25 (*n* = 3/n.a.)	< 0.01
	Lesinski et al. [4]^a^	0.78 (*n* = 10/17)	0.85 (*n* = 22/28)	> 0.05	0.91 (*n* = 3/5)	1.15 (*n* = 11/13)	> 0.05	0.85 (*n* = 27/40)	0.61 (*n* = 3/3)	> 0.05	0.80 (*n* = 47)		
	Moran et al. [26]^a^	0.91 (*n* = 7/13)	0.47 (*n* = 11/12) and 1.02 (*n* = 4/5)	> 0.05	–			0.73 (*n* = 22/30)			0.73 (*n* = 22/30)		
	Moran et al. [21]^a^	–			–				0.57 (*n* = 14/17)				
	Slimani et al. [23]^a^ CMJ	0.41 (*n* = 2/2)	0.69 (*n* = 13/15)	> 0.05	–			0.79 (*n* = 12/14)			0.65 (*n* = 13/16)		
	Slimani et al. [23]^a^ SJ	− 0.54 (*n* = 1/1)	0.95 (*n* = 9/9)	< 0.05	–			0.89 (*n* = 10/10)			0.80 (*n* = 8/10)		

*n.a.* not applicable, *n* number of included studies / intervention groups, **p** significance leve, *SJ* squat jump

^a^ Between-subject standardised mean difference

^b^Within-subject standardised mean difference

^c^Traditional resistance training; CMJ = Countermovement jump

^d^Power training (e.g., plyometric training)

**Table 4:** Summary of the findings (effect sizes = standardised mean differences) of the sub-group analyses regarding the effects of different resistance training types on measures of muscle strength and muscle power in healthy children and adolescents

Outcome	Study	Resistance training type			Traditional resistance training type		
		Traditional resistance training	Plyometric training	*p*	Machine-based training	Free weights training	*p*
Muscle strength	Behm et al. [20]^a^	1.14 (*n* = 37/44)	0.16 (*n* = 3/4)	n.a.	–		
	Behringer et al. [18]^b^	1.12 (*n* = 42/69)			0.93 (*n* = 31)	1.31 (*n* = 13)	> 0.05
	Lesinski et al. [4]^b^		0.39 (*n* = 4/5)		0.36 (*n* = 3/3)	2.97 (*n* = 2/4)	< 0.001
Muscle power	Behm et al. [20]^b^	0.52 (*n* = 39/47)	0.69 (*n* = 41/54)	n.a.	–		
	Collins et al. [5]^b^ vertical jump	0.41 (*n* = 17/25)			–		
	Collins et al. [5]^b^ SJ	0.73 (*n* = 9/10)			–		
	Lesinski et al. [4]^b^		0.81 (*n* = 16/25)		1.45 (*n* = 3/3)	0.90 (*n* = 3/5)	> 0.05
	Moran et al. [26]^b^		0.73 (*n* = 22/30)		–		
	Moran et al. [21]^b^		0.57 (*n* = 14/17)		–		
	Slimani et al. [23]^b^ CMJ	0.65 (*n* = 14/17)			–		
	Slimani et al. [23]^b^ SJ	0.80 (*n* = 9/10)			–		

*CMJ* countermovement jump, *n.a.* not applicable, *n* number of included studies/intervention groups, *p* significance level, *SJ* Squat jump, traditional resistance training comprises machine-based resistance training and free weights training

^a^Within-subject standardised mean difference

^b^Between-subject standardised mean difference

In terms of maturation status, sub-group analyses indicated large effects of RT in prepubertal participants (0.81 ≤ SMD ≤ 0.91) and medium-to-large effects in mid-/postpubertal participants (0.61 ≤ SMD ≤ 1.91) on muscle strength and power (Table 3). For muscle strength, Behringer et al. [18] found that prepubertal children gained significantly (*p* < 0.05) less muscle strength following RT (SMD = 0.81) compared to mid-/postpubertal adolescents (SMD = 1.91). Nevertheless, for muscle power, Lesinski et al. [4] as well as Collins et al. [5] did not find significant maturity-specific RT effects.

In terms of chronological age, sub-group analyses indicated medium-to-large effects of RT in children (0.57 ≤ SMD ≤ 1.35) and adolescents (0.69 ≤ SMD ≤ 0.91) on muscle strength [4, 17, 19] as well as small-to-large effects of RT on muscle power [4, 23, 26] in children (0.41 ≤ SMD ≤ 0.91) and adolescents (0.47 ≤ SMD ≤ 1.02) (Table 3). With the exception of Slimani et al. [23], there is no meta-analysis available that reported statistically significant effects of chronological age (i.e., children versus adolescents) for measures of muscle strength or muscle power [4, 5, 18, 26]. Nevertheless, the analysis of continuous moderator variables as reported by Behringer et al. [16] revealed a statistically significant (*p* < 0.05) negative correlation (*r* = − 0.25) between chronological age and the magnitude of effect sizes for motor skills (i.e., combined jumping, running, and throwing). This indicates that RT could be more beneficial in younger participants.

In terms of the sex variable, sub-group analyses indicated medium-to-large effects of RT in boys (0.72 ≤ SMD ≤ 1.21) and girls (0.54 ≤ SMD ≤ 1.42) on muscle strength [4, 17, 18] (Table 3). Further, the effects of RT on muscle power [4, 21, 23, 26] turned out to be medium-to-large for boys (0.73 ≤ SMD ≤ 0.89) and medium for girls (0.57 ≤ SMD ≤ 0.61) (Table 3). Collins et al. [5] found that boys (SMD = 0.84) compared with girls (SMD = 0.21) gained significantly (*p* < 0.01) more muscle power following RT. It has to be noted though that no other meta-analysis reported a statistically significant sex-specific effect of RT on muscle strength or muscle power [4, 18]. Payne and colleagues [17] did not examine the level of statistical significance.

In terms of expertise level, the included meta-analyses indicated medium-to-large effects of RT on muscle strength and muscle power in young athletes (i.e., trained children and adolescents) (0.65 ≤ SMD ≤ 1.09) [4, 5, 23, 26] (Table 3). Collins et al. [5] conducted a sub-group analysis regarding participants’ expertise level. They found that trained (SMD = 0.95) compared to untrained children and adolescents (SMD = 0.25) gained significantly (*p* < 0.01) more muscle power following RT.

In terms of RT type, the included meta-analyses indicated large effects of traditional RT (SMD = 1.12) [18] as well as small effects of plyometric training (SMD = 0.39) [4] on muscle strength (Table 4). Other meta-analyses indicated medium-to-large effects of traditional RT (0.41 ≤ SMD ≤ 0.80) [5, 23] or plyometric training (0.57 ≤ SMD ≤ 0.81) [4, 21, 26] on muscle power (Table 4).

Moreover, regarding the type of traditional RT, sub-group analyses indicated small-to-large effects (0.36 ≤ SMD ≤ 0.93) of machine-based RT and large effects (1.31 ≤ SMD ≤ 2.97) of free weights RT on muscle strength [4, 18]. Even though Behringer et al. [18] did not find statistically significant differences between traditional RT types in trained and untrained children and adolescents, Lesinski et al. [4] reported that free weights RT (SMD = 2.97) resulted in statistically significant larger gains in muscle strength (*p* < 0.001) compared to machine-based RT (SMD = 0.36) in trained children and adolescents.

## Discussion

This systematic umbrella review aimed to provide an overview of the effects of RT on proxies of physical fitness in healthy children and adolescents. The main findings of this umbrella review are: (1) RT has medium-to-large effects on measures of muscle strength, small-to-large effects on muscle power, small-to-medium effects on linear sprint speed, a medium effect on agility/change-of-direction speed, small-to-large effects on throwing performance, and a medium effect on sport-specific performance; (2) there are few consistent findings from the included meta-analyses regarding the moderating effects of age, maturation, sex, expertise level, and/or RT type on muscle strength and muscle power, and (3) the included meta-analyses are of low-to-moderate methodological quality and the presented evidence is of low or even very low quality.

### Effects of Resistance Training on Physical Fitness in Healthy Youth

This umbrella review indicates that RT interventions can enhance physical fitness in children and adolescents beyond a level which is not exclusively achievable from growth and maturation. We found that the effects of RT on measures of muscle strength and power were small-to-large in magnitude, with small-to-medium effects for secondary outcomes including linear sprint speed, agility/change-of-direction speed, and sport-specific performance. Therefore, effect sizes vary according to the respective outcome measure. The lower effects of RT on secondary outcomes can most likely be explained by the principle of training specificity [28] which suggests that the greatest strength gains occur at or near the training velocity.

### Effects of Moderating Factors Such as Age, Maturation, Sex, Expertise Level, Resistance Training Type

In terms of chronological age (Table 3), the reported sub-group analyses of the meta-analyses of Moran et al. [26], Lesinski et al. [4], and Behringer et al. [18] were unable to show any statistically significant age-related effects of RT on measures of muscle strength and muscle power. Notably, Behringer et al. [16] observed a statistically significant negative correlation (*r* = − 0.25) between chronological age and the magnitude of effect sizes for motor skills performance (i.e., jumping, running and throwing) in their meta-analysis. These authors proposed that younger children might experience a greater effect of RT on motor skills. In accordance with this finding, Slimani et al. [23] observed in their meta-analysis that adolescents compared with children improved their squat jump performance significantly more following RT. The observed differences in findings between the included meta-analyses could be due to differences in literature research strategies (e.g., different search syntax, inclusion criteria, or year of literature research) and applied methods. Taken together, the included meta-analyses consistently reported no chronological age-related effects of RT on measures of muscle strength. For measures of muscle power, the included meta-analyses revealed no consistent findings with regards to the moderating effects of age on RT-related training effects.

Unlike chronological age, maturation is not a linear process. Skeletal, sexual and somatic maturation in children differ individually in timing and tempo which is why there is often a discrepancy between chronological and biological age (i.e., maturation) among youths [29–32]. In terms of the maturation status (Table 3), a meta-analysis [18] found that prepubertal children gained significantly less muscle strength following RT (SMD = 0.81) compared with mid-/postpubertal adolescents (SMD = 1.91). For measures of muscle power, two meta-analyses [4, 5] were unable to identify any maturation-related effects of RT. Taken together, maturity seems to be an important moderating variable with regards to RT-related effects on muscle strength. While strength gains in prepubertal children mostly occur due to neural adaptations, additional morphological adaptations may explain the increased effects of RT in mid-/postpubertal adolescents [3].

In terms of the moderating factor sex (Table 3), Behringer et al. [18] were unable to identify significant sex-related effects of RT on measures of muscle strength in their meta-analysis. Further, Lesinski et al. [4] could not find statistically significant sex-specific effects of RT on muscle power in their meta-analysis. Nevertheless, a recent meta-analysis of Collins et al. [5] found that boys gained significantly more muscle power following RT (SMD = 0.84) compared with girls (SMD = 0.21). This finding has to be interpreted with caution because only one original study with girls was included in the respective sub-group analysis. Taken together, our findings suggest that boys and girls show similar RT-related improvements on measures of muscle strength. Controversial results exist in the available meta-analyses on sex-related effects of RT on measures of muscle power. Therefore, this research question must be investigated in future studies. Over the past years, more RT studies with youth reported data on the maturational status of the included participants. However, there is currently no meta-analysis available that examined ‘biological maturity’ as a moderating variable in its overall and/or sex-specific sub-group analyses. Accordingly, we commend pursuit of such research in the future.

In terms of the moderating factor expertise level, Behm et al. [20] and Collins et al. [5] conducted sub-group analyses in their meta-analyses regarding the role of expertise level (i.e., trained vs. untrained) in RT-related performance gains in youth (Table 3). While Behm et al. [20] could not find a moderating effect of expertise level on RT-related performance improvements in muscle strength and power in youth, Collins et al. [5] observed significantly larger effects on muscle power (i.e., squat jump performance) in trained (SMD = 0.95) compared with untrained youth (SMD = 0.25). Yet, findings from Collins et al. [5] have to be interpreted with caution due to the limited number of included original studies which examined the effects of RT on muscle power in untrained children and adolescents (*n* = 3). Accordingly, it can be argued that the inconsistent findings of the two meta-analyses are due to differences in the applied methods (i.e., within- versus between-subject SMD). Taken together, the available scientific evidence showed no robust results for the role of expertise level on RT related improvements in youth muscle power.

In terms of the moderating effects of the type of RT (i.e., traditional RT vs. plyometric training; Table 4), the included meta-analyses showed that traditional RT produced large effects on muscle strength [18], while plyometric training caused small effects on muscle strength [4]. Thus, it seems that traditional RT causes larger gains in muscle strength compared to plyometric training including high-velocity and muscle power exercises. This was confirmed by Behm et al. [20] who conducted a meta-analysis on the effects of traditional RT versus power training (i.e., plyometric training) on muscle strength in youth by aggregating within-subject SMDs. These authors found that traditional RT induced large effects while power training induced only trivial effects on measures of muscle strength. However, these findings are limited due to the low number of included studies that investigated the effects of power training on muscle strength (*n* = 3) as well as due to the applied statistical approach (i.e., calculation of within-subject SMDs). Of note, within-subject SMDs are biased because of regular growth and maturation-related performance enhancements in children and adolescents. In terms of muscle power, Behm et al. [20] reported slightly higher effect size magnitudes for jump performance following power training (within-subject SMD = 0.69) compared with traditional RT (within-subject SMD = 0.53). Notably, both pooled within-subject SMDs were of medium magnitude, which is why the evidence for larger effectiveness following power training is limited. Taken together, the above-mentioned meta-analyses indicate that with reference to the principle of training specificity [28], effect size magnitudes vary according to the respective outcome measure and RT type. This means that the greatest strength gains occur at or near the respective training velocity [28]. For instance, exercises with high-velocity movements such as plyometrics specifically enhance performances in movements with similar force–velocity profiles such as vertical and/or horizontal jumps.

Furthermore, in terms of the type of traditional RT (machine-based RT versus free weights), the meta-analysis of Behringer et al. [18] did not reveal statistically significant differences between the effects of free weight versus machine-based RT on measures of muscle strength in children and adolescents. However, another meta-analysis [4] found that free weights RT (SMD = 2.97) resulted in significantly larger gains in muscle strength compared with machine-based RT (SMD = 0.36) in young athletes. Each of the observed RT types has specific benefits and limitations [33, 34]. Supervised machine-based RT may allow a more stable performance of movements (e.g., lifts) which is why they can be considered an adequate learning tool for children and adolescents to start RT. Supervised RT using free weights allows to perform the full range-of-motion which better mimics sports-specific movements [33, 34]. It might be possible that children and adolescents who have reached a certain expertise level (i.e., trained youth), may better respond to free weights RT, compared with the general youth population. Taken together, the available scientific evidence shows no robust results for the factor type of traditional RT on muscle strength.

A clear limitation of meta-analyses is that they synthesize results from heterogenous original studies but do not consider important differences across the included original studies in terms of exercise programme variables, testing methods, and other factors. Therefore, the consideration of comparative intervention studies is needed that assess the effects of moderating factors such as age, maturation, sex, expertise level, or RT type on measures of physical fitness in children and adolescents while holding other variables constant. In this regard, Peitz et al. [35] recently conducted a systematic review of 75 comparative studies on the effects of traditional RT and plyometric training on physical fitness in youth aged 6–18 years. Their findings indicate that maturity-related effects are different following traditional RT versus plyometric training, with the former showing smaller and the latter showing larger effects in prepubertal children [35]. Further, there seems to be no sex-specific effects of traditional RT on physical fitness outcomes [35]. However, the impact of sex on plyometric training adaptions is unresolved [35]. Prepubertal boys and girls seem to respond similarly, while midpubertal boys show larger gains in jump performance compared with girls [35]. Finally, comparative studies [35] show that both traditional RT and plyometric training are effective. However, moderating factors such as maturity and sex appear to modulate the effects following traditional RT and plyometric training differently [35].

### Quality of the Included Meta-analyses

The methodological quality of the included meta-analyses can be classified as moderate-to-low. For the assessment of the methodological quality, Shea et al. [11] recommend that individual AMSTAR2 item ratings should not be combined to create an overall score. Users should consider the potential impact of an inadequate rating for each item independently. With the exception of Collins et al. [5], none of the included meta-analyses registered their protocol. Furthermore, only Lesinski et al. [4] explained the choice of study design for inclusion. Finally, none of the included meta-analyses provided a list of excluded studies (that were read in full text form) to justify their exclusion or reported sources of funding for the original (primary) studies. It might be possible that due to word/table/figure restrictions and/or the absence of databases for supplement materials, authors were unable to submit all information they had extracted from the primary research. Nevertheless, it might also be possible that authors were unaware of the importance of these methodological quality characteristics.

All included meta-analyses were classified as presenting low or very low quality of evidence. This might partly be due to under-reported GRADE items that also downgraded the quality of evidence. More specifically, risk and publication bias were often not reported. Because of the lack of meta-analyses with moderate or high quality of evidence, we are unable to draw conclusions as to whether future research (i.e., meta-analyses) with high quality of evidence might change the strengths of this recommendation.

### Suggestions for Future Research

To strengthen preliminary findings regarding the effects of RT on a wide range of physical fitness outcomes, future research should investigate the effects of RT on secondary outcomes (e.g., agility/change-of-direction, throw, sport-specific performance) as well. Given that the sub-group analyses of the included meta-analyses with regards to the moderators age, maturation, and sex are mostly based on a low number of included studies, future research should especially focus on examining the effects of RT in prepubertal children and girls, irrespective of their maturational status. Furthermore, future research should document participants’ biological maturity status as well as distinguish between the different types of RT. Of note, biological maturity can easily be assessed through the maturity offset method as introduced by Mirwald et al. [10] or by recording Tanner stages. These variables should be included as moderators in sex-specific sub-group analyses. Finally, research with high methodological quality and high quality of evidence should be conducted in the future.

### Strengths and Methodological Limitations

This umbrella review presents findings on the highest level of the medicine evidence pyramid regarding the effects of RT on proxies of physical fitness in healthy children and adolescents. Furthermore, this umbrella review ensured a high-level synthesis of potentially moderating variables and addressed the methodological quality and the quality of evidence. Finally, this umbrella review identified current gaps in the literature to make suggestions for future research.

A limitation of this umbrella review is the rather low number of meta-analyses (*N* = 14) which were eligible for inclusion. Another limitation is the low methodological quality and the (very) low quality of evidence of the included meta-analyses. Some of the assessed AMSTAR2 as well as GRADE criteria are under-reported or under-represented. In addition, it is important to acknowledge that even if the meta-analyses investigated similar research questions they showed methodological differences in search strategies and selection criteria as well as with regards to the applied analytical approach. It has to be noted that some primary studies were included across multiple meta-analyses while others were not. Consequently, the general weight of the single primary studies can be different. Furthermore, findings regarding the observed sub-group analyses of the moderating factors of RT mostly showed no consistent and robust results and, thus, must be interpreted with caution.

## Conclusion

This systematic umbrella review proved that RT has the potential to enhance proxies of physical fitness in healthy children and adolescents beyond a level achievable from growth and maturation. We found that the effects of RT on measures of muscle strength and muscle power were small-to-large in magnitude, with small-to-medium effects for secondary outcomes including linear sprint, agility/change-of-direction, and sport-specific performances.

Our findings further indicate that there are few consistent effects of potentially moderating factors such as ‘chronological age’, ‘maturation’, ‘sex’, ‘expertise level’, and ‘RT type’ on measures of muscle strength and muscle power in healthy children and adolescents across the included meta-analyses. Preliminary findings suggest that ‘maturation’ (i.e., prepubertal < mid-/postpubertal) as well as ‘type of RT’ (i.e., traditional RT > plyometric training) moderate the effects of RT on muscle strength while ‘chronological age’ and ‘sex’ appear not to. Whether the factors ‘expertise level’ and ‘type of traditional RT’ have an impact on muscle strength cannot be elucidated based on the available data. Furthermore, preliminary findings suggest that the potentially moderating variables ‘maturation’, ‘sex’, and ‘type of RT’ do not modulate RT-related adaptions in youth’ muscle power. Whether ‘chronological age’, ‘expertise level’, and ‘type of traditional RT’ have an impact on muscle power is currently unresolved. Due to the limited amount of original research on specific sub-groups (e.g., girls, children, prepubertal youth), the findings of the included meta-analyses and, thus, of this umbrella review, regarding the effects of the moderating factors (e.g., sex, maturation) on RT on muscle strength and power have to be interpreted with caution. However, the benefits of safely performed and supervised RT are now irrefutable. RT should be used extensively in schools and should be embedded into PE curricula globally.

### Data Availability

All data are provided in the article and the Electronic Supplementary Material.

## Electronic supplementary material

Below is the link to the electronic supplementary material.

Supplementary material 1 (DOCX 18 kb)

Supplementary material 2 (DOCX 16 kb)
