# Supplementary material for: Effects of Resistance Training on Physical Fitness in Healthy Children and Adolescents: An Umbrella Review
Source: Sports Med. 2020 Aug 5;50(11):1901–28. doi: 10.1007/s40279-020-01327-3 (PMC7575465; doi:10.1007/s40279-020-01327-3)
Supplement: Supplementary file 1 — Supplementary material 1 (DOCX 18 kb) [file 40279_2020_1327_MOESM1_ESM.docx]

**Electronic Supplementary Material Table S1**: Results of the assessment of the methodological quality of the included meta-analyses using AMSTAR 2 (A measurement Tool to Assess systematic reviews)

| Meta-analysis | AMSTAR 2 items | | | | | | | | | | | | | | | | Score |
| --- | --- | --- | --- | --- | --- | --- | --- | --- | --- | --- | --- | --- | --- | --- | --- | --- | --- |
|  | **1** | **2** | **3** | **4** | **5** | **6** | **7** | **8** | **9** | **10** | **11** | **12** | **13** | **14** | **15** | **16** |  |
| Asadi et al. [27] | Yes | No | No | Partial yes | Yes | Yes | No | Partial yes | No | No | No | No | No | No | No | No | 25% |
| Behm et al. [20] | Yes | No | No | Partial yes | Yes | No | No | Partial yes | No | No | Yes | No | No | No | No | Yes | 31% |
| Behringer et al. [18] | Yes | No | No | Partial yes | No | No | No | No | Yes | No | Yes | No | Yes | No | Yes | No | 34% |
| Behringer et al. [16] | Yes | No | No | Partial yes | No | No | No | No | Yes | No | Yes | No | Yes | Yes | Yes | Yes | 47% |
| Collins et al. [5] | Yes | Yes | No | Yes | Yes | Yes | No | Partial yes | Yes | No | No | Yes | Yes | Yes | Yes | Yes | 72% |
| Falk et al. [19] | Yes | No | No | No | No | No | No | No | No | No | Yes | No | No | No | No | No | 13% |
| Harries et al. [24] | Yes | No | No | No | Yes | No | No | No | Yes | No | Yes | Yes | No | No | No | Yes | 38% |
| Lesinski et al. [4] | Yes | No | Yes | Partial yes | Yes | No | No | Partial yes | Yes | No | Yes | Yes | No | No | No | Yes | 50% |
| Moran et al. [26] | Yes | No | No | Partial yes | No | Yes | No | No | No | No | Yes | No | No | Yes | No | Yes | 34% |
| Moran et al. [25] | Yes | No | No | Partial yes | No | Yes | No | Partial yes | No | No | Yes | No | No | Yes | No | Yes | 38% |
| Moran et al. [22] | Yes | No | No | Partial yes | No | Yes | No | Partial yes | No | No | Yes | No | No | No | No | Yes | 31% |
| Moran et al. [21] | Yes | No | No | Partial yes | No | Yes | No | Partial yes | No | No | Yes | No | No | Yes | No | Yes | 38% |
| Payne et al. [17] | Yes | No | No | No | No | No | No | No | No | No | No | No | No | No | No | No | 6% |
| Slimani et al. [23] | Yes | No | No | Partial yes | No | Yes | No | Partial yes | No | No | Yes | No | No | No | Yes | Yes | 38% |

Legend: 1 = Word research question and inclusion criteria according to PICOS (population, intervention, comparator, outcome, study design), 2 = Establish methods prior to the conduct of the meta-analyses (written protocol), 3 = Explain the choice of study design for inclusion, 4 = Use comprehensive literature search strategy, 5 = Perform study selection in duplicate, 6 = Perform data extraction in duplicate, 7 = Provide a list of excluded studies to justify the exclusion, 8 = Describe the included studies in detail, 9 = Assess the risk of bias, 10 = Reported sources of funding for included studies, 11 = Use appropriate method for statistical combination of results, 12 = Assess the potential impact of risk of bias for included studies, 13 = Account for risk of bias while interpreting/discussing the results, 14 = Explain/discuss any heterogeneity, 15 = Assess publication bias and discuss its impact on the results, 16 = Report potential sources of conflict of interest and describe any funding

**COMPLIANCE WITH ETHICAL STANDARDS**

**Funding**

This review is part of the research project ‘Resistance Training in Youth Athletes’ that was funded by the German Federal Institute of Sport Science (ZMVI1-081901 14-18, ZMVI4-081901/20-23).

**Conflicts of Interest**

Melanie Lesinski, Michael Herz, Alina Schmelcher and Urs Granacher declare that they have no conflicts of interest relevant to the content of this review.

**Authorship Contributions**

ML, MH, AS, UG extracted, analysed and interpreted the data. ML, MH, AS, and UG wrote the manuscript.
