# Supplementary material for: Effects of Resistance Training on Physical Fitness in Healthy Children and Adolescents: An Umbrella Review
Source: Sports Med. 2020 Aug 5;50(11):1901–28. doi: 10.1007/s40279-020-01327-3 (PMC7575465; doi:10.1007/s40279-020-01327-3)
Supplement: Supplementary file 2 — Supplementary material 2 (DOCX 16 kb) [file 40279_2020_1327_MOESM2_ESM.docx]

**Electronic Supplementary Material Table S2:** Results of the assessment of the quality of evidence for each outcome of the included meta-analyses using GRADE (Grading of Recommendations Assessment, Development and Evaluation)

| **Meta-Analysis** | **Outcome** | **GRADE items** | | | | | **Quality of the evidence** |
| --- | --- | --- | --- | --- | --- | --- | --- |
|  |  | **Risk of bias** | **Inconsistency** | **Indirectness** | **Imprecision** | **Publication bias** |  |
| Asadi et al. [27] | Change-of-direction speed | Not reported (-1) | Not reported (-1) | Neutral | Serious (-1) | Not reported (-1) | Very low |
| Behm et al. [20] | Muscle strength | Not reported (-1) | PT: Neutral  ST: Very serious (-2) | Neutral | Serious (-1) | Not reported (-1) | PT: Very low  ST: Very low |
|  | Muscle power | Not reported (-1) | PT: Serious (-1)  ST: Neutral |  | Neutral | Not reported (-1) | PT: Very low  ST: Low |
|  | Linear speed | Not reported (-1) | PT: Neutral  ST: Neutral |  | Serious (-1) | Not reported (-1) | PT: Very low ST: Very low |
| Behringer et al. [18] | Muscle strength | Serious (-1) | Not reported (-1) | Neutral | Neutral | Very serious (-2) | Very low |
| Behringer et al. [16] | Jump | Serious (-1) | Not reported (-1) | Neutral | Not reported (-1) | Serious (-1) | Very low |
|  | Linear speed |  | Not reported (-1) |  | Not reported | Serious (-1) | Very low |
|  | Throw |  | Not reported (-1) |  | Not reported | Serious (-1) | Very low |
|  | Combined motor performance |  | Neutral |  | Neutral | Serious (-1) | Low |
| Collins et al. [5] | Vertical jump | Serious (-1) | Neutral | Neutral | Neutral | Serious (-1) | Low |
|  | Squat jump | Serious (-1) | Serious (-1) |  | Neutral | Serious (-1) | Very low |
|  | Standing long jump | Serious (-1) | Neutral |  | Neutral | Serious (-1) | Low |
|  | Linear speed | Serious (-1) | Neutral |  | Neutral | Serious (-1) | Low |
|  | Throw | Serious (-1) | Neutral |  | Neutral | Serious (-1) | Low |
| Falk et al. [19] | Muscle strength | Not reported (-1) | Not reported (-1) | Neutral | Not reported (-1) | Not reported (-1) | Very low |
| Harries et al. [24] | Vertical jump | Serious (-1) | Serious (-1) | Neutral | Neutral | Not reported (-1) | Very low |
| Lesinski et al. [4] | Muscle strength | Serious (-1) | Very serious (-2) | Neutral | Neutral | Not reported (-1) | Very low |
|  | Vertical jump | Serious (-1) | Serious (-1) |  | Neutral | Not reported (-1) | Low |
|  | Linear sprint | Serious (-1) | Neutral |  | Neutral | Not reported (-1) | Low |
|  | Change of direction speed | Serious (-1) | Serious (-1) |  | Neutral | Not reported (-1) | Very low |
|  | Sport-specific performance | Serious (-1) | Serious (-1) |  | Neutral | Not reported (-1) | Very low |
| Moran et al. [26] | CMJ | Not reported (-1) | over all: Serious (-1) | Neutral | Neutral | Not reported (-1) | Very low |
| Moran et al. [25] | Muscle strength | Not reported (-1) | over all: Very serious (-2) | Neutral | Neutral | Not reported (-1) | Very low |
| Moran et al. [22] | Muscle strength | Not reported (-1) | Neutral | Neutral | Neutral | Not reported (-1) | Low |
| Moran et al. [21] | Vertical jump | Not reported (-1) | Serious (-1) | Neutral | Neutral | Not reported (-1) | Very low |
| Payne et al. [17] | Muscle strength and endurance | Not reported | Serious (-1) | Neutral | Not reported | Not reported (-1) | Very low |
| Slimani et al. [23] | CMJ | Not reported | Serious (-1) | Neutral | Neutral | Neutral | Low |
|  | Squat jump | Not reported | Serious (-1) |  | Neutral | Neutral | Low |

Legend: PT = power training (e.g., plyometric training), ST = traditional resistance training

**COMPLIANCE WITH ETHICAL STANDARDS**

**Funding**

This review is part of the research project ‘Resistance Training in Youth Athletes’ that was funded by the German Federal Institute of Sport Science (ZMVI1-081901 14-18, ZMVI4-081901/20-23).

**Conflicts of Interest**

Melanie Lesinski, Michael Herz, Alina Schmelcher, and Urs Granacher declare that they have no conflicts of interest relevant to the content of this review.

**Authorship Contributions**

ML, MH, AS, UG extracted, analysed and interpreted the data. ML, MH, AS, and UG wrote the manuscript.
